# Influence of embedding Cu nano-particles into a Cu/SiO_2_/Pt structure on its resistive switching

**DOI:** 10.1186/1556-276X-8-156

**Published:** 2013-04-08

**Authors:** Chih-Yi Liu, Jyun-Jie Huang, Chun-Hung Lai, Chao-Han Lin

**Affiliations:** 1 Department of Electronic Engineering, National Kaohsiung University of Applied Sciences, No.415, Chien Kung Road, Kaohsiung, 807, Taiwan; 2 Department of Electronics Engineering, National United University, No.1, Lienda, Miaoli 360, Taiwan

**Keywords:** Cu nano-particle, Resistive switching, SiO_2_, 73.50.-h, 73.40.Rw, 73.61.Ng

## Abstract

Cu nano-particles (Cu-NPs) were embedded into the SiO_2_ layer of a Cu/SiO_2_/Pt structure to examine their influence on resistive switching characteristics. The device showed a reversible resistive switching behavior, which was due to the formation and rupture of a Cu-conducting filament with an electrochemical reaction. The Cu-NPs enhanced the local electric field within the SiO_2_ layer, which caused a decrease in the forming voltage. During successive switching processes, the Cu-NP was partially dissolved, which changed its shape. Therefore, the switching voltages were not reduced. Moreover, the Cu-NPs caused a non-uniform Cu concentration within the SiO_2_ layer; thus, the Cu-conducting filament should be formed in a high Cu concentration region, which improves switching dispersion. The Cu-NPs within the SiO_2_ layer stabilize the resistive switching, resulting in a larger switching window and better endurance characteristics.

## Background

Portable electronic products are common in daily life. A requirement of portable electronic products is low power consumption. Non-volatile memory (NVM) can retain information without a power supply, which is suitable for portable products. Flash memory is currently the mainstream product in NVM devices. However, it will eventually reach its physics limitations with continuous scaling, which causes retention degradation and serious reliability issues. Therefore, numerous novel devices for replacing flash memory have been proposed. Among these devices, the resistive random access memory (RRAM) with a simple metal/insulator/metal structure shows a reversible resistive switching behavior [1]. The device resistance can switch between a high-resistance state (HRS) and a low-resistance state (LRS) using dc voltages or pulses. Numerous materials with various resistive switching behaviors, such as NiO [2], HfO_2_[3], SrZrO_3_[4], and SiO_2_[5] have been proposed. Several switching mechanisms such as electrochemical [6], thermochemical [7], and valance change effect [8] have been proposed to explain the various switching behaviors. However, resistive switching is unstable, which may cause operating issues [9,10]. Several methods such as doping [11], process optimization [12], interface control [13], and embedding nano-particles [14-16] have been adopted to improve the switching dispersion in various switching behaviors. All studies used inactive materials for their embedded nano-particles when examining their effect on switching behavior [14,17]. The inactive nano-particles enhanced the local electric field within the resistive layer, which decreased the operating voltages and improved the switching dispersion [17].

Pt nano-particles were embedded into the resistive layer in our previous study [18] to examine their influence on the resistive switching of an electrochemical-based RRAM device. The improvement of the switching dispersion resulted from the enhancement of the local electric field within the resistive layer. An electrochemical-based RRAM device generally has an active electrode and a counter inert electrode. The active metal is partially dissolved and acts as a cation supplier. The cations migrate in an electric field through the resistive layer and are reduced at the inert cathode. Thereafter, a metallic filament grows toward the anode and connects the two electrodes. The growth of the conducting filament is through the preferred ionic drift path within the resistive layer. Thermadam et al. proposed that the Cu concentration of the resistive layer influenced the resistive switching behavior [19]. The influence of the embedded nano-particles of an active metal on electrochemical-based RRAM has not been examined. The nano-particles of active metals within the resistive layer may change the distribution of the local electric field and cation supply. In this study, Cu nano-particles (Cu-NPs) were embedded into a Cu/SiO_2_/Pt structure to examine the role of Cu-NPs on resistive switching. The forming voltage was reduced in the Cu-NP sample; this was due to the enhancement of the local electric field. The improvement of switching dispersion may be caused by the non-uniform Cu concentration in the SiO_2_ layer.

## Methods

Four-inch p-type silicon wafers were used as substrates. After a standard Radio Corporation of America cleaning, a 200-nm-thick SiO_2_ layer was thermally grown in a furnace to isolate the Si substrate. Thereafter, a 5-nm Ti layer and a 100-nm Pt layer were deposited by an electron-beam evaporator to form a Pt/Ti/SiO_2_/Si structure. The Pt layer was adopted as the bottom electrode. A 20-nm SiO_2_ layer was deposited using radio frequency (rf) sputtering at room temperature on the Pt electrode. A 10-nm Cu layer was deposited with a thermal evaporator at room temperature on the 20-nm SiO_2_ layer to examine the influence of Cu-NPs. Thereafter, a rapid thermal annealing was performed at 600°C for 5 s in a nitrogen ambient to form the Cu-NPs. A 20-nm SiO_2_ layer was subsequently deposited on the Cu-NPs. Furthermore, the 150-nm Cu top electrodes patterned by a metal mask were deposited using a thermal evaporator coater to fabricate a Cu/Cu-NP embedded SiO_2_/Pt device (Cu-NP sample). The area of the device was approximately 5×10^−5^ cm^2^. A Cu/SiO_2_/Pt device (control sample) was additionally fabricated without the Cu-NPs formation procedures for comparison purposes. The cross section of the Cu-NP sample was observed with a high-resolution transmission electron microscopy (HRTEM, TEM-3010, JEOL, Ltd., Tokyo, Japan). The distribution of the Cu concentration within the structure was analyzed using energy-dispersive X-ray spectroscopy (EDX). Electrical measurements were performed using an HP 4155B semiconductor parameter analyzer (Hewlett-Packard Company, Palo Alto, CA, USA) at room temperature. The bias voltage was applied on the Cu top electrode while the bottom electrode was grounded. The applied voltage was swept with a step of 20 mV, and the compliance current was 1 mA.

## Results and discussion

**Figure 1:**
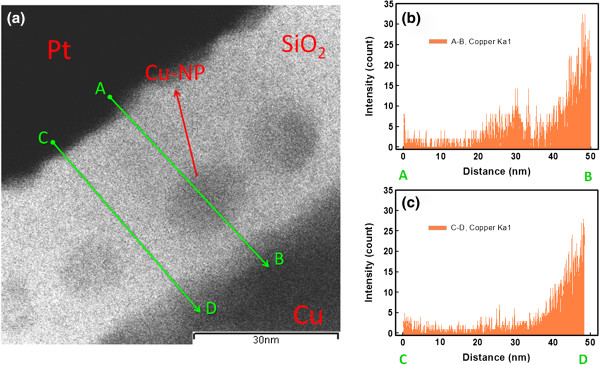
**Cu concentrations within SiO**_**2 **_**layer along different paths.** (**a**) HRTEM cross-sectional image of a Cu/Cu-NP embedded SiO_2_/Pt sample. (**b**) Energy-dispersive X-ray spectroscopy (EDX) result along line A-B. (**c**) Energy-dispersive X-ray spectroscopy (EDX) result along line C-D.

Figure 1a shows the HRTEM cross-sectional image of the pristine Cu-NP sample. The Cu-NPs formed within the SiO_2_ layer. The size of the Cu particles was approximately 10 nm. Figure 1b,c shows the EDX line scans of the Cu-NPs sample along the indicated lines in Figure 1a. Figure 1b shows the EDX line scan through a Cu particle (line A-B), and Figure 1c shows the EDX line scan through a region without a Cu-NP (line C-D). In general, the Cu concentration gradually decreased from the Cu top electrode to the Pt bottom electrode, which indicates that the Cu atoms diffused from the Cu top electrode into the SiO_2_ layer. As shown in Figure 1b, an obvious Cu peak was observed in the middle of the SiO_2_ layer, indicating that a Cu-NP was located within the SiO_2_ layer. As shown in Figure 1c, a small peak was observed in the middle of the C-D line, which was caused by the lateral Cu diffusion from the nearest Cu-NPs. Moreover, the Cu-NPs may cause vertical diffusion during the fabrication procedures. Therefore, the A-B line region had a higher Cu concentration than the C-D line region. The Cu atoms were non-uniformly distributed in the SiO_2_ layer.

**Figure 2:**
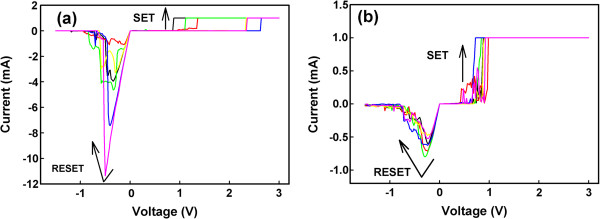
**Influence of Cu**-**NPs on reversible switching current**-**voltage characteristics.** (**a**) Resistive switching characteristics of the Cu/SiO_2_/Pt structure. (**b**) Resistive switching characteristics of the Cu/Cu-NP embedded SiO_2_/Pt structure.

**Figure 3:**
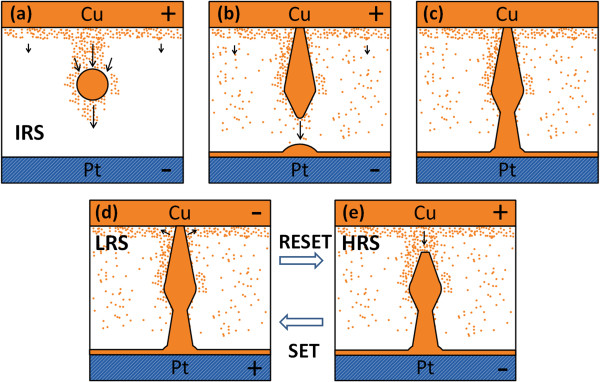
**Schematic illustration of switching operation of the Cu**-**NP sample.** (**a**) Initial stage of the forming process. (**b**) Middle stage of the forming process. (**c**) After the forming process. (**d**) The RESET process. (**e**) The SET process.

Figure 2 shows the resistive switching characteristics of the two samples. Only six successive switching cycles were illustrated in each figure, and each cycle was painted with different colors. The two samples showed reversible resistive switching behaviors. The device current abruptly increased from an initial resistance state to a LRS when a large positive voltage (forming voltage) was applied onto a pristine device, which is referred to as the forming process (not shown). Thereafter, the device current abruptly decreased when a certain negative voltage was applied to the device, switching it to a HRS, which is referred to as the RESET process. Furthermore, the device current abruptly increased at a certain positive voltage (SET voltage), switching it to a LRS, which is referred to as the SET process. During the forming process and SET process, a compliance current of 1 mA was adopted to prevent current damage. The device current can reversibly switch between a LRS and a HRS using dc voltages under different polarities. The resistance states can maintain the same values for more than 10^4^ s, which indicate that the devices are suitable for NVM applications. Because of the switching behavior, device structure, and our previous study [18], the Cu filament model with the electrochemical reaction [6] was adopted to explain the switching mechanism. Figure 3 shows the schematic illustration of switching operation of the Cu-NP sample. Figure 3a,b,c shows the forming process. The embedded Cu-NP causes a larger Cu concentration and enhances the local electric field near itself in the vertical direction. Due to the larger electric field and larger Cu concentration, a Cu filament is formed through the Cu-NP. The Cu cations migrate from the top electrode to deposit on the Cu-NP. Due to charge equilibrium during the forming process, the Cu cations are also dissolved from the bottom part of the Cu-NP and then migrate to deposit on the bottom electrode. Finally, a Cu conducting filament is formed through the Cu-NP (Figure 3c). The shape of Cu-NP is changed during the forming process. Two necks are formed within the Cu conducting filament. Figure 3d,e shows the SET and RESET processes in the Cu-NP samples. Due to the structure geometry, the electric field near the Cu/SiO_2_ interface (upper neck) is larger during the RESET process; thus, rupture/formation of the Cu filament occurs at the Cu/SiO_2_ interface.

**Figure 4:**
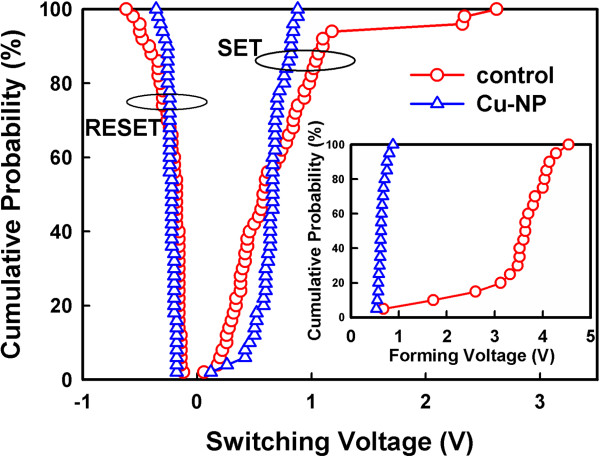
**Influence of Cu**-**NPs on the operating voltages.** Statistical results of SET and RESET voltages of the control and the Cu-NP samples. The inset shows statistical results of forming voltages.

**Figure 5:**
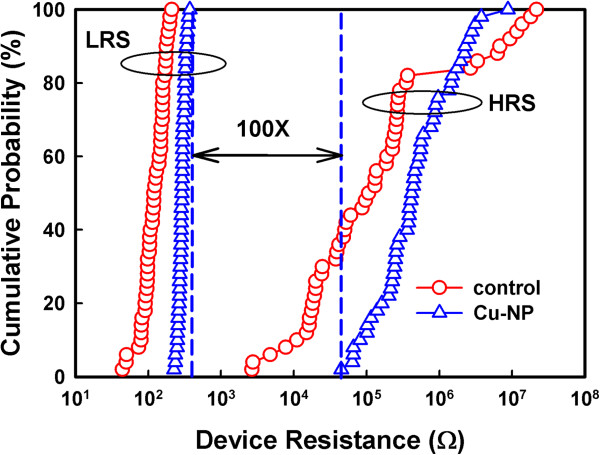
**Influence of Cu**-**NPs on the different resistance states.** Statistical results of HRS and LRS resistances of the control and the Cu-NP samples.

The statistic results of operating voltages are shown in Figure 4. The inset shows the forming voltages of the two samples. The forming voltage of the Cu-NP sample was approximately 0.6 V, but the control sample was approximately 3.6 V. The switching dispersion was improved by the Cu-NPs. The Cu-NPs enhanced the local electric field within the SiO_2_ layer, reducing the forming voltage.The Cu-conducting filament preferentially formed in a large electric field region, which additionally reduced the switching dispersion. Moreover, the non-uniform Cu concentration within the SiO_2_ layer should improve the switching dispersion. Therefore, the Cu-NP sample had better characteristics in the forming process than the control sample. The magnitudes of the SET voltage and RESET voltage of the two samples were identical. The switching dispersion was improved by the Cu-NPs. In our previous study [18], the embedded Pt-NPs improved resistive switching and decreased the magnitude of the operating voltage. However, the effect of the Cu-NPs on resistive switching was significantly different from that of the Pt-NPs. The resistive switching was caused by the rupture and formation of a Cu-conducting filament through the dissolution and electrodeposition of Cu atoms. During the RESET process, the Pt-NPs did not dissolve and maintained their shape to enhance the local electric field. The enhancement of the electrical field was dependent on the curvature radius of the particles. The portion of the Cu-NP with a smaller curvature radius had a larger electrical field, which could be dissolved into Cu cations. Therefore, the Cu-NPs were partially dissolved during the RESET process and their shape was altered. The Cu-NPs did not maintain their particle shape to enhance the local electrical field to decrease the magnitude of the operating voltages. Therefore, no non-uniform electrical field decreased the switching dispersion. Figure 1 indicates that the Cu atoms were not uniformly distributed in the SiO_2_ layer. Moreover, the partially dissolved Cu-NPs act as an ion supplier in the vertical direction through Cu-NPs. The SiO_2_ layer with higher Cu concentration assisted the formation of the Cu filament [19]. The Cu filament forms in a high Cu concentration region. Therefore, the non-uniform Cu concentration by Cu-NPs within the SiO_2_ layer improved the switching dispersion. Figure 5 shows the statistic distribution of the LRS and HRS resistances of the two samples. The resistance variations of the Cu-NP sample were smaller than those of the control sample, which were caused by the stable switching of the Cu-NPs. The switching margin of the Cu-NP sample was more than two orders, which provided the possibility of a multilevel design.

**Figure 6:**
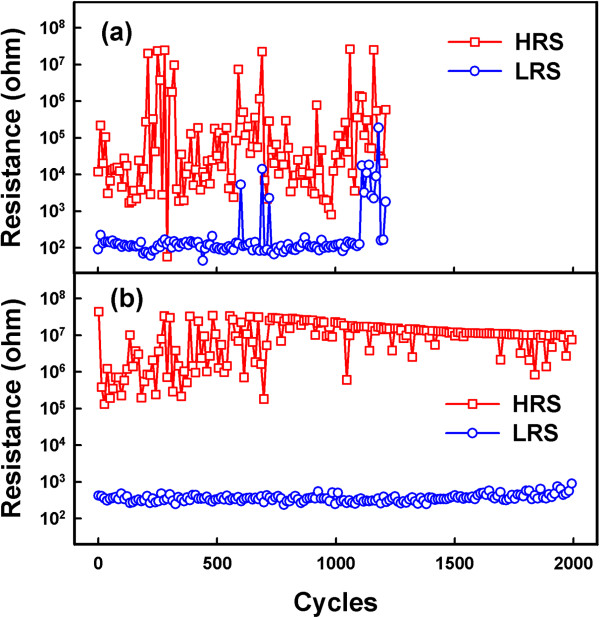
**Influence of Cu**-**NPs on the endurance behaviors.** (**a**) Endurance characteristics of the control sample. (**b**) Endurance characteristics of the Cu-NP sample.

Figure 6 shows the endurance characteristics of the control sample and the Cu-NP sample using dc voltage sweeping. The endurance of the control sample was only 1,200 cycles, and the resistance states showed a large dispersion. Several soft errors were observed, which may cause operating issues. The endurance of the Cu-NP sample was more than 2,000 cycles, and the resistance states showed a small dispersion. The switching margin of the Cu-NP sample was more than 100, which provided a large sensing margin. The Cu-conducting filament was ruptured and formed through these Cu-NP regions, which stabilized the switching process and improved the endurance characteristics.

## Conclusions

Cu-NPs were embedded into the SiO_2_ layer of the Cu/SiO_2_/Pt structure to examine their influence on resistive switching behavior. The Cu-NPs enhanced the local electrical field during the forming process, which decreased the magnitude of the forming voltage and improved the switching dispersion. However, during the subsequent switching processes, the Cu-NPs were partially dissolved and their particle shape was altered; thus, the local electrical field was not enhanced by the Cu-NPs and did not decrease the magnitude of the operating voltages. The Cu-NP fabrication process and partial dissolution of the Cu-NPs in the switching process caused non-uniform Cu concentration within the SiO_2_ layer. Non-uniform Cu distribution caused the Cu-conducting filament to form in a high Cu concentration region, which improved the switching dispersion. The Cu-NPs stabilized the resistive switching, and subsequently improved endurance characteristics.

## Abbreviations

Cu-NPs: Cu nano-particles; EDX: Energy-dispersive X-ray spectroscopy; HRS: High resistance-state; HRTEM: High-resolution transmission electron microscopy; LRS: Low resistance-state; NVM: Non-volatile memory; RRAM: Resistive random access memory

## Competing interests

The authors declare that they have no competing interests.

## Authors’ contributions

CYL designed the experiment, participated in the result analysis, and wrote the paper. JJH and CHL (Lin) prepared the devices and carried out the TEM analyses and electrical measurements. CHL (Lai) assisted in the electrical measurements and result analysis. All authors read and approved the final manuscript.

## Authors’ information

CYL is an associate professor at the Department of Electronic Engineering, National Kaohsiung University of Applied Sciences, Taiwan. JJH is a master student at the Department of Electronic Engineering, National Kaohsiung University of Applied Sciences, Taiwan. CHL (Lai) is an associate professor at Department of Electronic Engineering, National United University, Taiwan. CHL (Lin) is a master student at the Department of Electronic Engineering, National Kaohsiung University of Applied Sciences, Taiwan.
